# RNA motifs, RNA structure, and motif context analyzed by RNAanalyzer^3^

**DOI:** 10.1093/nar/gkag392

**Published:** 2026-04-30

**Authors:** Aman Akash, Johannes Balkenhol, Chunguang Liang, Kathi Zarnack, Thomas Dandekar

**Affiliations:** Chair of Bioinformatics, Biocenter, Am Hubland, University of Würzburg, 97074 Würzburg, Germany; Chair of Bioinformatics, Biocenter, Am Hubland, University of Würzburg, 97074 Würzburg, Germany; Chair of Bioinformatics, Biocenter, Am Hubland, University of Würzburg, 97074 Würzburg, Germany; Institute of Immunology, Jena University Hospital, Friedrich-Schiller-University Jena, 07743 Jena, Germany; Chair of Bioinformatics, Biocenter, Am Hubland, University of Würzburg, 97074 Würzburg, Germany; Chair of Bioinformatics, Biocenter, Am Hubland, University of Würzburg, 97074 Würzburg, Germany

## Abstract

RNAanalyzer^3^ (“RNA analyzer cubic”; https://rnaanalyzer.bioapps.biozentrum.uni-wuerzburg.de) substitutes the frequently consulted current RNAanalyzer webserver (https://rnaanalyzer-old.bioapps.biozentrum.uni-wuerzburg.de). RNAanalyzer^3^ is free/open via the secure HTTPS protocol, with example data, help and tutorial, web-link to results, and rich data output. We combine a general detailed structure analysis with motif analyses. It accepts either a single plain-text nucleotide sequence or batch submission in FASTA format, which can be pasted or uploaded as a FASTA file. Our tool (i) has up-to-date software and operating systems, (ii) combines diverse RNA motif analyses with RNA structure prediction, (iii) puts found motifs into structural context, and (iv) offers dedicated tools for probing RNA–protein binding interactions. RNAanalyzer^3^ links motif searches to Rfam and miRNA search to miRbase. It focuses on structural features first, looks for stem-loops, hairpins, and specific enrichment regions such as stem-GG pairs, plus AU-rich regions with their locations for easier identification, while providing structural context and interactive RNA structure visualization. A tabulated overview shows all RNA features including structure details, coding potential, untranslated regions (UTRs, including Shine–Dalgarno sequences, Kozak sequences, and polyadenylation signals), transfer RNA (tRNA), microRNA (miRNA), long noncoding RNA (lncRNA), *trans*-splicing motifs, iron response elements (IRE), riboswitches, small nuclear ribonucleoprotein (snRNP) motifs, and spliceosomal Sm-sites.

## Graphical Abstract

**Graphical Abstract:**
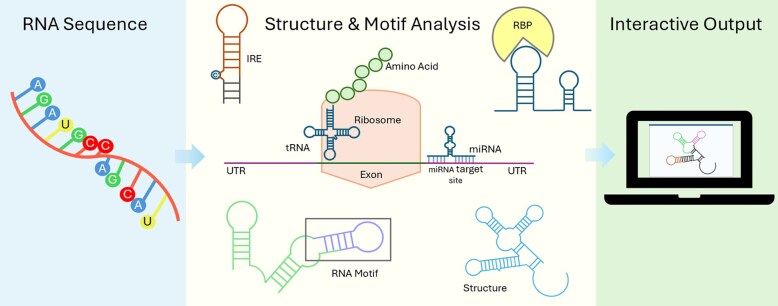

## Introduction

RNA biology has gained significant traction in the past decade, with a particular focus on its regulatory functions [1, 2]. Intensive research in RNA structure and motifs has revealed various functions of noncoding RNAs, which actively regulate transcriptional and post-transcriptional changes [3, 4]. RNA structure plays a large role in its functions and drives its interaction, influencing regulatory features [5]. Similarly, RNA motifs can be utilized to annotate functions of a given RNA sequence based on sequence conservation, structural features, or a combination of both [6, 7]. However, the growing complexity of RNA biology has led to tools and databases becoming specific in checking for a specific type of feature [8–11].

RNAanalyzer (2003) was a general-purpose RNA toolbox that gave users an overview of the sequence and structural features of a given RNA sequence [12]. However, due to the vast number of discoveries in RNA biology as seen in updated RNA-centric sources like Rfam and miRBase, analyses of RNA motifs have been expanded beyond the potential of the previous web server [13–15]. To keep up with these changes, we present RNAanalyzer^3^, speak: “RNA analyzer cubic,” a free web server which is open to all users without any login requirements. It is based on RNA structural context principles and provides users with a broad overview of any RNA sequence. RNAanalyzer^3^ replaces the old RNAanalyzer [16] with an integrated structural and context analysis, including stem-loops and specific RNA motif searches. It also includes RNA-binding protein site scans, coding potential analysis, miRNA and miRNA target analysis, polyA signal check, including polyA tail, and more. RNAanalyzer^3^ is built to be species-agnostic, which helps in identifying features for a broader range of organisms. Moreover, RNAanalyzer^3^ annotates and integrates all important features discovered directly onto the RNA structure with a detailed annotation map using a specific subroutine built upon VARNA [17] and optionally FORNA [18] from ViennaRNA tools.

### Materials and methods

Developed in Perl, RNAanalyzer^3^ utilizes CGI module for rich web output combined with JavaScript for visualization. The Perl backend makes it possible to integrate various tools and parse their output for a more user-friendly end result. RNAanalyzer^3^ is a structure-based tool, and it starts by establishing the structure of the given RNA sequence and then performs various scans to determine the function or type of RNA [19].

RNAanalyzer^3^ accepts sequences in FASTA format and is programmed to accept up to five sequences with a maximum sequence length of up to 20 000 nucleotides. Some features, like folding calculation and structure visualization, are limited to <5000 nucleotides due to higher loading times. For pseudoknot and suboptimal secondary folding predictions, the limit is lower, up to 2500 and 1500 nucleotides, respectively, to negate long runtimes. Example sequences are also given for various types of RNA features with a quick example button to test the web server. A job ID is assigned to each submission, which can be later used to retrieve the previous results. We also allow the users to download the results as a PDF or text file. The details of the feature scans and routines can be found in the Supplementary Information.

The updated results page shows all the results from the selected and built-in scans in tabular output with a collapsible header, which can be used to keep the results page much simpler and tidier to use. For regulatory elements recognized, there are database crosslinks (if external) or own literature and cross-reference descriptions (IRE, *trans*-splicing).

**Figure 1.**
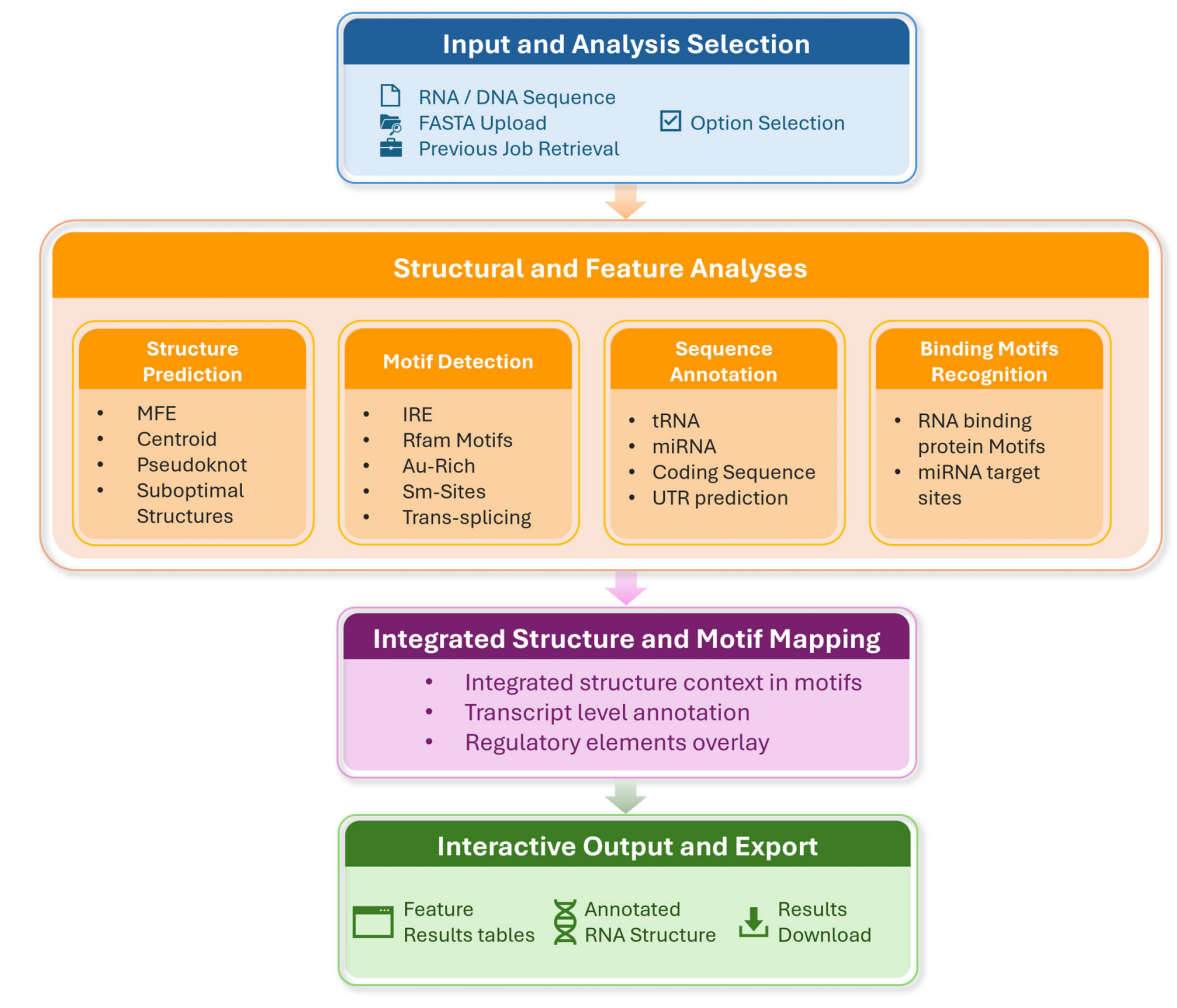
Workflow of RNAanalyzer^3^. The input sequence is analyzed for identifying various structures and sequence features. The final results are displayed in their representative interactive tables and annotated RNA structure, along with the feature table.

The structures for the whole sequence and motifs are interactive and can be panned and zoomed. The updated results page can also be bookmarked and retrieved later for revisiting results. The workflow of RNAanalyzer^3^ is illustrated in Fig. 1 with a more extensive process chart provided in Supplementary Fig. S1.

## Results

The built-in Perl routines, carefully combined with external tools, give a rich output that provides users with an extensive overview of the RNA structure, motifs, and other sequence elements in any given nucleotide sequence. Carrying on the same idea as the older RNAanalyzer, RNAanalyzer^3^ provides an integrated RNA feature analysis, which can detect important structural features and regulatory motifs. Up-to-date and comprehensive databases are used for the type of motif and/or RNA detection, coupled with the relevant tools. Apart from the standard MFE secondary structure, RNAanalyzer^3^ allows prediction of centroid and pseudoknot prediction and visualization using RNAfold [19] and Ipknot (v1.1.0) [20], respectively. Some internal modules, like IRE, still use the MFE structure for structural confirmation of the predicted motifs, whereas the embedded pseudoknot secondary structure prediction is limited to visualization. RNA folding is limited due to long processing times. Nevertheless, the user can process up to five sequences of 20 000 nucleotides each, allowing for a total of 100 000 nucleotides to be analyzed per session. 

The stem-loop detection algorithm has been refined to improve biological relevance. The updated method recognizes stem-loops with a minimum of three unpaired bases, along with a minimum of three paired bases. It now includes checks for terminal hairpins and creates stacks of the detected stem-loops to avoid recounting. Bulge detection is also added to avoid breaking or recounting the stem-loops. This updated routine is also incorporated into various other routines to add structural context, such as UTR prediction, structured region scan, and riboswitch prediction, specifically where the structure can be informative [19, 21, 22]. Sequence base composition is also included in RNAanalyzer^3^ results, helpful in detecting AU or GC content, which can help in determining the stability of the sequence [23].

Trans-splicing and iron-responsive element (IRE) search in the sequence are performed by separate Perl routines originally made for RNAanalyzer [24, 25]. IRE search has been updated to include the new functionally validated IRE in *Pfn2* [14, 26]. The list of functionally validated IREs recognized by RNAanalyzer^3^ is listed in Supplementary Table S5. The updated IRE module produces as output possible structure(s) for the identified IRE motif with a quality measure based on their minimum folding energy (MFE). When compared, the internal, strict rule-based IRE module of RNAanalyzer^3^ showed more specificity and precision for canonical IREs as compared to the SIREs 3.0 webserver, which allows predictive IRE recognition. The benchmarking results for validated IREs against the SIREs 3.0 web server are shown in Supplementary Table S7. The predicted IRE structures can also be viewed with an interactive viewer. Other Perl routines scan for Cstf elements, catalytic sites, and AU-rich regions. The output of old routines was updated to match the output of the newer and latest routines in the updated RNAanalyzer^3^. In the new RNAanalyzer^3^, structural context is always taken into account, either during motif detection or when assembling and mapping the different outputs to the full RNA structure. We also validated the main functional routines, including the updated IRE module, *trans*-splicing, ARE, and Sm-site, using a targeted validation strategy where we compared the known positive sequence, random negatives, and also controlled and random mutations. The details of the internal modules are listed in Supplementary Table S2, with their targeted validation separately in Supplementary Table S3. We give detailed quantitative benchmarks in Supplementary Table S8 for IRE, Supplementary Table S9 for Au-rich elements, Supplementary Table S10 for *trans*-splicing, and Supplementary Table S11 for Sm-site. For the known instances of a specific motif, rules are defined for stem-loops, energy, and sequence motifs, and the motif is recognized once these rules are fulfilled. This allows RNAanalyzer^3^ to detect motifs in all organisms, or for instance, also in pristine metagenomics data.

Motif detection is central to understanding RNA function and type. In RNAanalyzer^3^, motif identification is performed for several specific motifs, like the above examples, followed by a general motif scan using the Rfam database, a comprehensive database for RNA motif families [13]. RNAanalyzer^3^ uses covariance models, which, apart from considering sequence conservation, also check for structural similarities [27]. A general motif scan helps minimize overlooking of RNA motifs but can result in duplicate results. A structure visualization for the detected Rfam motif is also built into RNAanalyzer^3^, with a link to the Rfam database for more information on that motif. Riboswitch prediction now adds structural context with functional insights for common riboswitches. We also allow users to retrieve the raw output from all the external tools, allowing a more detailed outlook when required.

Coding potential of an RNA transcript describes if the RNA can be translated into a protein and which regions of the RNA constitute the coding sequence (CDS). Coding RNAs were previously thought to be translated without having regulatory functions, but this paradigm has recently changed, as noncoding RNAs, such as long noncoding RNA (lncRNA), are now found to code for proteins [28]. However, coding regions of RNA are still not associated with regulatory function. Keeping this in mind, it is important to determine if an RNA sequence can code for a protein or not. In RNAanalyzer^3^, it can be predicted in two ways: First, the default prediction of coding potential is performed by coding potential calculator 2 (CPC2) [29], a species-agnostic tool, and also by Augustus, which is an automated pipeline for gene annotation. Augustus utilizes more accurate species-specific models to scan the given sequence and can also predict the UTR for some species. Users can choose Augustus if their sequence is from one of the available model species, which includes human, *Caenorhabditis elegans, Escherichia coli, Arabidopsis thaliana, Saccharomyces cerevisiae, Drosophila melanogaster*, and *Danio rerio* (zebrafish) [30]. To detect UTRs for all other species, we developed two routines that can facilitate the prediction of UTRs in a species-agnostic manner. The routine first captures the predicted CDS and exons in the given sequence and then utilizes them to predict UTRs. When UTR is not predicted due to missing exon boundaries, the second routine checks for polyA inference, which can be used to predict the 3′ UTR, which is important in post-transcriptional gene expression. If the sequence is deemed to be noncoding, then a structural scan is performed to identify sequence parts with heavy structural features. This simple structural scan can also help in highlighting unannotated or novel regions in the RNA structure. In addition to structural analysis, coding-potential prediction is also used to determine whether a transcript qualifies as a lncRNA. A transcript is typically classified as a lncRNA if it is >200 nucleotides, lacks an open reading frame (ORF) or contains an ORF <300 nucleotides, and exhibits a weak Kozak sequence when an ORF is present. Due to the absence of a definitive rule, these parameters can still be utilized to predict lncRNAs [31, 32]. Recognized bigger motifs are also presented on the annotated structure, which further adds structural context to the identified motifs across all modules.

miRNA target scan in RNAanalyzer^3^ is performed on the predicted 3′ UTR sequence. It scans the whole sequence when no UTR is predicted. This helps maintain biological significance and reduces overall runtime, especially in large sequences. miRNA binding sites in the 3′ UTR are a crucial post-transcriptional regulation that can lead to translational repression or degradation of mRNA [35]. Apart from the miRNA binding scan in the 3′ UTR, RNAanalyzer^3^ also performs an RNA-binding protein (RBP) site scan on the whole sequence to predict sites where RNA-binding proteins can bind. RNA-binding proteins can play a vital role in post-transcriptional regulation, which includes splicing, transport, and decay, among others [36, 37]. Supplementary Table S1 summarizes the external tools integrated in RNAanalyzer^3^, along with our contributions. Supplementary Table S4 provides an overview of the specific structural features analyzed, compared, and contextualized by RNAanalyzer^3^.

Following the analysis, the nucleotide positions of motifs, secondary structures, UTRs, and coding regions are stored in arrays, which are used to annotate the structure with motif-specific color codes, taking all structural features and the whole RNA structure into account. The annotated structure is interactive and allows zoom and closer inspection using VARNA [17], with an added option to use FORNA [18] as an alternative when the VARNA visualization is cluttered or unclear. This can benefit users in pinning the structure of the important features identified in the sequence. The sequence is also numbered every 50 nucleotides, which can be helpful to identify the exact locations of the feature. The annotated structure is accompanied by a feature table to make the identification in the structure easier for the user. To our knowledge, no other web tool yet annotates a structure like RNAanalyzer^3^ and can provide important structural insights in the sequence as well as the features. A brief comparison of the various aspects of similar web servers is laid out in Table 1, stating the gaps filled by RNAanalyzer^3^, highlighting its functionality when compared to existing web servers. A more detailed comparison is listed in Supplementary Table S6, which categorizes the contribution of RNAanalyzer^3^ and compares it with the listed webservers.

**Table 1.** Comparison of widely used RNA web tools, summarizing functions, core methods, and gaps mentioned, plus added advantages of RNAanalyzer^3^ in comparison

Tool	Scope/primary function	Core methods/models	Gaps addressed/Advantages of RNAanalyzer^3^	Ref.
RNAanalyzer (2003)	Integrated web server to identify various RNA elements	Overview of regulatory RNA elements	We extend the older web server with broader motif scans, cleaner interactive output, and annotated structure visualization.	[12]
RegRNA 3.0	Broad identification of regulatory RNA motifs/elements	Integrates motif libraries and updated detectors across numerous categories	We integrate sequence-based motif scans with structural context, predicted CDS, and UTR for an extensive RNA sequence overview rather than just motif annotation. It also allows the user an easier interpretation of overlapping motifs.	[16]
SIREs 3.0	Specialized prediction of IREs	Updated IRE models; improved scoring and multi-mode input workflow	We provide canonical IRE identification within a broader multi-feature RNA analysis and is not limited to single motif detection.	[11]
RNAcentral	Comprehensive ncRNA sequence database combining many expert resources	Aggregation from 60 + expert DBs; 2D structure integration	We offer a transcript analysis workflow that combines structure-centered analysis, motif scanning, coding potential, annotated structure visualization, and is not limited to database-bound sequence annotation.	[33]
RNAfold Webserver (ViennaRNA Web)	Predict RNA secondary structure	Dynamic programming for MFE and RNA structure	We integrate secondary structure visualizations along with a motif detection framework.	[34]
RNAanalyzer^3^	RNA analysis that integrates structural features with diverse motif analyses, combined with coding potential	Unified structure + motif pipeline, coding-potential, and with UTR inference; cross-links to external databases	We provide an RNA sequence overview platform combining various feature scans along with structural representation and integration of various modules.	This study

### Routine use case

To check the performance of the updated RNAanalyzer^3^ and its capability to detect various features present in any RNA sequence, we required a mRNA with a distinct RNA motif combined with UTRs and CDS. The mRNA of Ferritin heavy chain 1 (*FTH1*) fits perfectly as it contains CDS, UTR, and an IRE. *FTH1* encodes for ferritin heavy chain protein which is important in iron metabolism and also plays a role in oxidative stress [38, 39]. It has also been identified as a marker in disease contexts like cancer and neurodegeneration, among others [40–42]. The sequence of *FTH1* was retrieved from NCBI with accession NM_002032.3 and was submitted to RNAanalyzer^3^ with default options.

**Figure 2.**
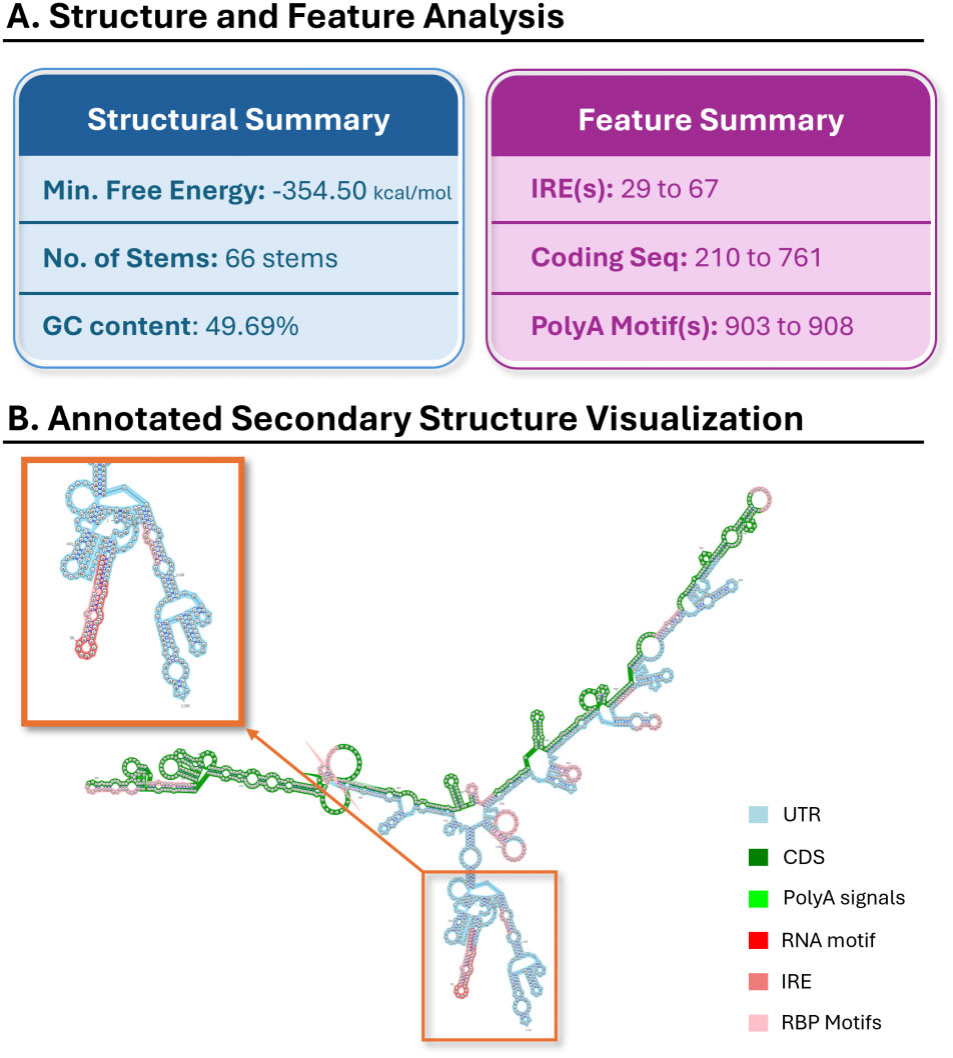
(**A**) Representative summary of the various structural and sequence features analyzed by RNAanalyzer^3^ in the FTH1 mRNA. The first table shows the structural features, and second table shows the motifs and sequence features. (**B**) Structure of the given RNA sequence annotated with color schemes for various identified features, including the IRE motif in the 5′ UTR.

RNAanalyzer^3^ recognized the IRE motif in the 5′ UTR and provided results with 3 possible alternate structures of the IRE motif. Figure 2A shows example structural features like folding energy, stem loops, GC content, etc., along with RNA sequence features detected like IRE motif, coding sequence, polyA sites, among others, in the *FTH1* mRNA transcript. To check if RNAanalyzer^3^ was able to correctly recognize IRE in the sequence, an IRE scan was also performed with SIREs 3.0. SIREs detected two possible IREs, but only one with high confidence, which matches the RNAanalyzer^3^ prediction (Supplementary Fig. S2). However, in SIREs output, only a predefined structure is provided for the identified IRE, which can differ from the actual folding as discussed earlier [11]. The element is also recognized with the general RNA motif scan, which can be used to check details of the motif family on the Rfam database [13]. We ran a similar scan with the new RegRNA 3.0 webserver, which was able to identify many functional motifs in the *FTH1* mRNA sequence (Supplementary Fig. S3), which, although comprehensive, can lead to convoluted results where UTR regions are detected overlapping the CDS sequence. This can be attributed to the independent scans performed by RegRNA, which are feature-rich but lack interconnected identification. To overcome this in RNAanalyzer^3^, much of the motif identification is limited to various regions like polyA signals, and miRNA target search in the 3′ UTR.

Apart from the motif recognition, RNAanalyzer^3^ was also able to predict the coding potential with a great degree of accuracy using the CPC2 module. The UTRs were also correctly inferred from the CDS boundaries, and the potential protein sequence was calculated from the detected exons.

Thus, the annotated structure of Ferritin heavy chain 1 (*FTH1*) mRNA highlights the recognized IRE in the 5′ UTR, as shown in Fig. 2B. The IRE can be identified with a coral color on the RNA structure from bases 29 to 67 in the 5′ UTR, resembling structure 2 identified in the IRE scan, which further shows that the RNA structure of motifs can vary in the folding of the whole sequence. The UTR regions are colored blue while the coding sequence is shown in green, extending from nucleotides 210 to 761. RNA-binding protein sites recognized by RNAanalyzer^3^ are annotated in salmon color and can be seen overlapping the UTRs and CDS. The UTRs and the CDS have numerous motifs overlapping their sequence at various places to facilitate the identification of regulatory motifs. A polyA tail was identified in the 3′ UTR, which can be seen forming a loop. Together, these annotations on the RNA provide a clear map of RNA motifs and the structural milieu of the *FTH1* mRNA transcript.

To further demonstrate the functional utility of RNAanalyzer^3^, we tested it with the tumor necrosis factor (*TNF*) mRNA transcript (NM_000594.4), which is known to have a densely regulated 3′ UTR. RNAanalyzer^3^ was able to infer the 3′ UTR and recognized many ARE motifs situated densely from 1341 to 1392, consistent with the well-established ARE-rich region of the *TNF* 3′ UTR [43]. The ARE results also overlapped with protein binding motifs specifically with *ZFP36 (TTP)* and *ELAVL (HUB)* family of proteins, which are known ARE binding protein classes, where *ZFP36* has also been reported to bind *TNF* [44]. The ARE hits and RBP motifs are given in the Supplementary Table S12. This illustrates that RNAanalyzer^3^ can integrate UTR context, regulatory motif identification, and RBP-associated annotation, providing a regulatory overview within one transcript-centered analysis.

Using these examples, we were able to prove the usability of RNAanalyzer^3^, which was able to correctly recognize not just the important regulatory features but also the coding features and UTRs in the provided transcript sequences.

The many structure-minded and motif-minded motifs analyzed imply numerous specific use cases:

- Search for the specific motif, put into a structural context. This main use case is optimized both for large-scale motif detection and detailed motif evaluation, and for summarizing the motif annotation directly mapped onto the RNA and all other motifs found.
- Genome annotation (up to 5000 nucleotides including folding, 20 000 nucleotides for sequence motif detection, up to five individual FASTA sequences), where we also detect tRNA (tRNAscan-SE [45]) and specific regulatory RNAs (both custom-written programs). Large-scale and diverse motif detection is furthermore done via Rfam and other databases such as miRbase and Miranda to detect miRNA.
- Evaluation of structural features together with a detailed look at the RNA motif involved, such as RNA–protein binding.
- Better phylogenetic understanding by looking for large phylogenetic distances in the structure of the RNA the user is interested in. For benchmarking of the distances inferred, users can utilize our phylogenetic toolbox ITS2 database (https://its2.bioapps.biozentrum.uni-wuerzburg.de/). Moreover, this includes also sequence-structure alignments (4SALE; https://4sale.bioapps.biozentrum.uni-wuerzburg.de/) for any RNA sequence family of interest [46, 47].

## Discussion

The rapid expansion in RNA biology has increased the demand for computational tools that can be employed to characterize the features of any given RNA sequence. Many such tools exist, for example, TargetScan, RBPMap, RNAfold webserver, SIREs 3.0, RegRNA 3.0, among others, which identify and/or predict specific features [8, 9, 11, 16, 19]. RNAanalyzer^3^ takes a more holistic approach by combining structural prediction, various motif scans, and coding potential analysis in one organized output, making it a versatile web server for RNA characterization. The innovation of our tool compared to current general RNA motif finders (e.g. RegRNA 3.0) is to assess both the specific RNA structure as well as the structural context of the whole RNA. Moreover, we have several self-developed structure-minded tools (for specific RNA motifs and structures such as IREs, *trans*-splicing RNA motifs) and scripts (for RNA folding, comparison of motifs found, and output context as well as processing).

The annotation of the features in a neatly presented interactive structure of any RNA sequence can be very helpful in providing sequence and structural insights. This, in combination with several local routines to identify features like *trans*-splicing, IREs, catalytic sites, and structures, could provide biological relevance to the function of the RNA. RNAanalyzer^3^ is species-agnostic, which makes its usage more friendly for working with a wider range of organisms. This is a key aspect, as many of the current webservers are species-specific and are limited to model organisms only. Moreover, several databases and resources provide species-specific data, which makes the implementation of species-agnostic features much more complex and even somewhat outdated in some cases. Overcoming these limitations will be another frontier we will focus on for future upgrades.

There are many valuable motif-specific tools like SIREs 3.0 [11], ARESite2 [48], and general motif webservers like RegRNA 3.0 [11, 16, 33] and alignment-based type of servers, e.g. RNACentral [11, 16, 33] for detailed and different types of RNA motif analysis. In extension and complementation of such analyses, RNAanalyzer^3^ provides a general look at the whole RNA structure, as well as all encoded motifs in their structural context with various features. For example, IRE identification is present in RNAanalyzer^3^; it looks for functional IRE motifs, taking structural features into consideration, having in this sense fewer over- or underpredictions compared to SIREs 3.0 [11]. RegRNA 3.0 is another web server that enables the identification of 26 regulatory features present in RNA sequences, but no structure or motif context is analyzed [16]. In extension, RNAanalyzer^3^ provides a general structure-aware analysis of the whole RNA molecule with coding and structural context for easier functional interpretation.

In biology, the full RNA molecule is important. This is more than a collection of motifs, and here RNAanalyzer^3^ brings the full context information to the user. We demonstrate this with two use cases. For instance, to understand the function of an IRE (iron-responsive element) depends on where it is positioned in the mRNA regarding its function: If it is in the 5´UTR, it regulates the translation of the following CDS (dependent on the binding of the IRE binding protein). If it is in contrast in the 3´UTR, it regulates the stability of the mRNA and has to occur in multiple instances. Hence, not looking at the context of the complete RNA would prevent understanding the specific function of the IRE identified, and here RNAanalyzer^3^ is a strong context visualizing tool. This is the case for many RNA regulatory elements; most function only in a specific mRNA context and a suitable section of the mRNA. The context thus also evaluates the prediction of the isolated element. To make this even clearer for the reader, we now have two use-case examples with *TNF* mRNA as a second example, having a known AU-rich UTR and its role in post-transcriptional regulation. RNAanalyzer^3^’s ability to predict the ARE elements in the 3′ UTR overlapping with RNA-binding protein motifs (RBP motifs), further stresses the functional use of our web server by putting everything into context, including RNA structure. In general, RNA Analyzer^3^ does rely on different tools which can be used separately, but it combines them in one context encompassing the major motif searches.

The various elements and their functions depend on their structural contexts and placement, especially regulatory elements. This is a key point for the usage of RNAanalyzer^3^.

Limitations and outlook: Building on its extensive structure-sequence analysis methods, RNAanalyzer^3^ allows several areas for further development. We will extend the regulatory motif identification capabilities of RNAanalyzer^3^ in a species-specific way, for example, splicing motifs, promoter recognition sites, among others. There is also a limitation regarding the structural representation of tRNAs, where folding can deviate from the canonical structure due to modified bases, which is not always properly accounted for by the integrated folding algorithm. We will hence further enhance structural feature detection, including tRNA folding as well as ribosome-binding sites, but also areas where our folding routines can excel, such as pseudo-knot detection. We will introduce an API to allow large-scale genome annotation.

In conclusion, we present RNAanalyzer^3^, a structure-minded and broad RNA motif analyzer and web server, which is simple to use, requiring no login, and provides a concise yet comprehensive overview of any given RNA sequence in its individual and overall RNA structure context. In addition, RNAanalyzer^3^ contains a series of specific and general RNA feature scans that can be selected by users and then presented in an easy-to-understand tabular output for each feature. Finally, it also annotates the important features on an interactive visualization of the RNA structure.

## Supplementary Material

gkag392_Supplemental_File

## Data Availability

RNAanalyzer^3^ is an open-access, free-to-access web server for bioinformatics analysis. The code for RNAanalyzer^3^ is public and is available on Zenodo with DOI 10.5281/zenodo.18196127. The license details for the tools used are present in the “License and Attributes” page of the web server, and the respective license information is also available in the repository.
