# Supplementary material for: RNA motifs, RNA structure, and motif context analyzed by RNAanalyzer3
Source: Nucleic Acids Res. 2026 Apr 30;54(W1):W188–95. doi: 10.1093/nar/gkag392 (PMC13355056; doi:10.1093/nar/gkag392)
Supplement: gkag392_Supplemental_File [file gkag392_supplemental_file.pdf]

Supplementary Information

Feature Scans:

Users can select which scans they would like to perform, which include IRE, tRNA scan, miRNA scan, RNA motif scan, RNA binding protein motif scan, trans-splicing, riboswitches, coding prediction, and miRNA target scan. Most of the scans are pre-selected, and others can be selected when needed, which includes miRNA target scan and coding potential with Augustus. Augustus is species-limited, and thus, when not chosen, coding prediction is done by a slightly modified CPC2, which can also give the strand information.

The IRE module of RNAanalyzer[3] detects canonical iron-responsive elements, which are well established in literature [1,2] as described in **supplementary table 5.** The module recognizes the IRE hairpins and rejects them if there are disruptions in the loop, bulge, or stem, which goes against the defined strict motif architecture [3,4]. Small mutations in the loop have been shown to be tolerated by the dedicated module. Another module is the trans-splicing module which also consists of a strict rule-based motif with added support elements around the recognized splice-core and Sm-site motif [3,5]. Any disruption in the splice-core or Sm-site leads to exclusion of the recognized sequence. RNAanalyzer[3] also looks for smaller motifs like Sm-site, Au-Rich elements using similar strict rule-based motif recognition. The Sm-site module recognizes snRNA binding motifs that match consensus motifs found in spliceosome small RNAs. While having high specificity, the module still reports any U-rich sequences. The Au-rich region recognition module is based on small characteristic AU-rich elements which boast good specific element recognition [3]. The internal modules are listed in **supplementary table 2**.

The internal modules are rule-based individual motif detectors, which were validated using targeted datasets with manually curated positives, random negatives, and random + intended mutations. This was performed to demonstrate the functionality of the updated and legacy modules, confirming the utility of the modules rather than establishing a large-scale benchmark performance. The benchmarking results are shown in **supplementary table 3** with individual targeted validation strategy datasets provided in **supplementary table 8** for IRE, **supplementary table 9** for Au-rich elements, **supplementary table 10** for trans-splicing, and **supplementary table 11** for Sm-site. There was no additional targeted validation of the CstF element, as this module is an example of a legacy rule-based component that was kept for consistency with previous versions.

Several in-built Perl routines utilize the folding, which is integrated in the scans for structural features like stems, hairpins, and motifs; iron-responsive elements, trans-splicing, etc. For other scans, we utilize widely used tools like CPC2 v0.1 or Augustus v3.5.0 for coding potential prediction [6,7], cmscan (Infernal v1.1.5) for RNA motif scan against the Rfam motif family database [8,9], tRNAscan-SE v2.0.12 for potential tRNA scan [10], yamtk v2.0.0 (yet another motif toolkit) based on FIMO from MEME suite [11,12], and hmmer v3.4 for microRNA scan with miRBase [13,14]. We ran a small internal benchmark using mRNA transcripts from random 100 human genes to compare the results of yamtk with FIMO (v5.5.8). The results showed yamtk was able to report all the motif hits which FIMO reported (2406 of 2411) with a high recall but also reported additional recognized short motifs. A custom Python wrapper is also built for miranda v3.3a for miRNA target scanning using mature miRNAs from miRBase [13,15]. The external tools described are listed in **supplementary table 1** with our extended advantages to their outputs.

To predict relevant biological inference from the sequence, RNAanalyzer[3] infers UTRs from exon boundaries predicted by CPC2 or utilizes UTR prediction by Augustus when chosen. The UTR sequences are then scanned for relevant motifs like SD and Kozak motif in 5' UTR and polyA tail and polyA signals in 3'UTR. In case UTR cannot be predicted, it scans for polyA signals and uses them to refine the UTR prediction. The predicted 3' UTR is also used for scanning the miRNA target sites to keep it more biologically relevant.

Every detected feature is stored in arrays, which are then used to annotate the interactive visualization of the RNA structure. The interactive visualization is achieved using VARNA [16] and optionally FORNA [17] from ViennaRNA tools and is accompanied by a legend and a feature table, which helps in identifying the locations of the features.

Technical Information

The inbuilt Perl routines are integrated with tools like ViennaRNA to analyze features like IRE, trans-splicing, etc. [18] The web server is compatible with all the modern web browsers, including Chrome, Edge, Firefox, etc. Web server performance is continuously monitored, with planned upgrades to ensure optimal run times.

The new batch submit module takes the input and sequentially analyzes it to present the user when the results are ready. The batch submit checks the input sequence for harmful or invalid input and sanitizes it if required. It also displays the maximum estimated runtime above the submission table, calculated based on total sequence length and selected run parameters.

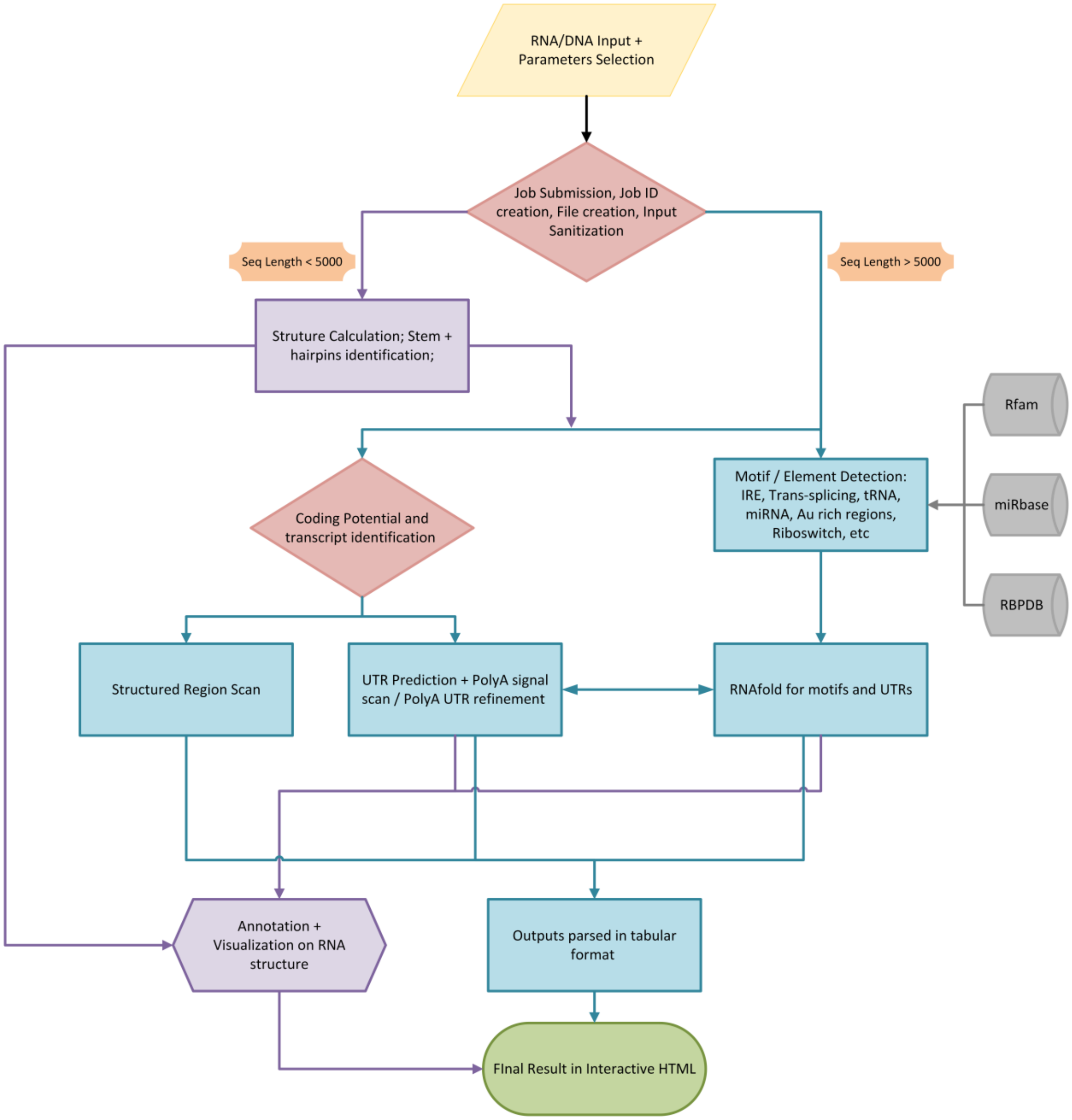

Supplementary Figure 1. *Workflow of RNAanalyzer[3]. RNA motif search (blue) is combined with different specific regulatory RNA element analysis, RNA-protein binding and RNA annotation (purple) with consideration of the structural context of individual motifs and whole RNA. For RNA > 5000 nucleotides, the RNA fold routine (purple) is skipped but all individual folds within the long RNA are still calculated. Total length limit is 20,000 nucleotides and a maximum of five FASTA sequences in one input.*

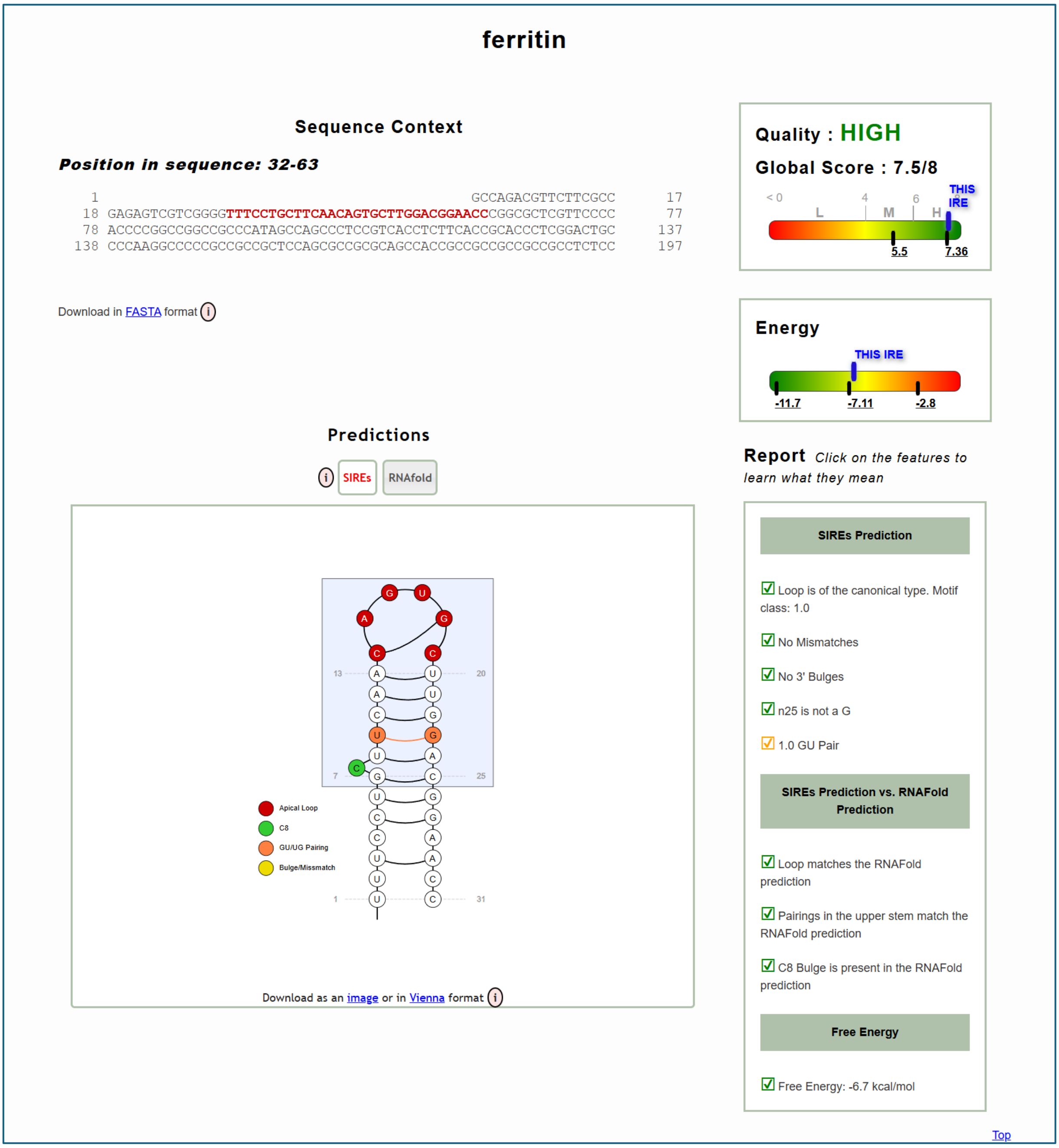

Supplementary Figure 2. *Iron-responsive element search for FTH1 mRNA using SIREs 3.0 webserver. The identified IRE with high quality matches the identification by RNAanalyzer*[3] *[19].*

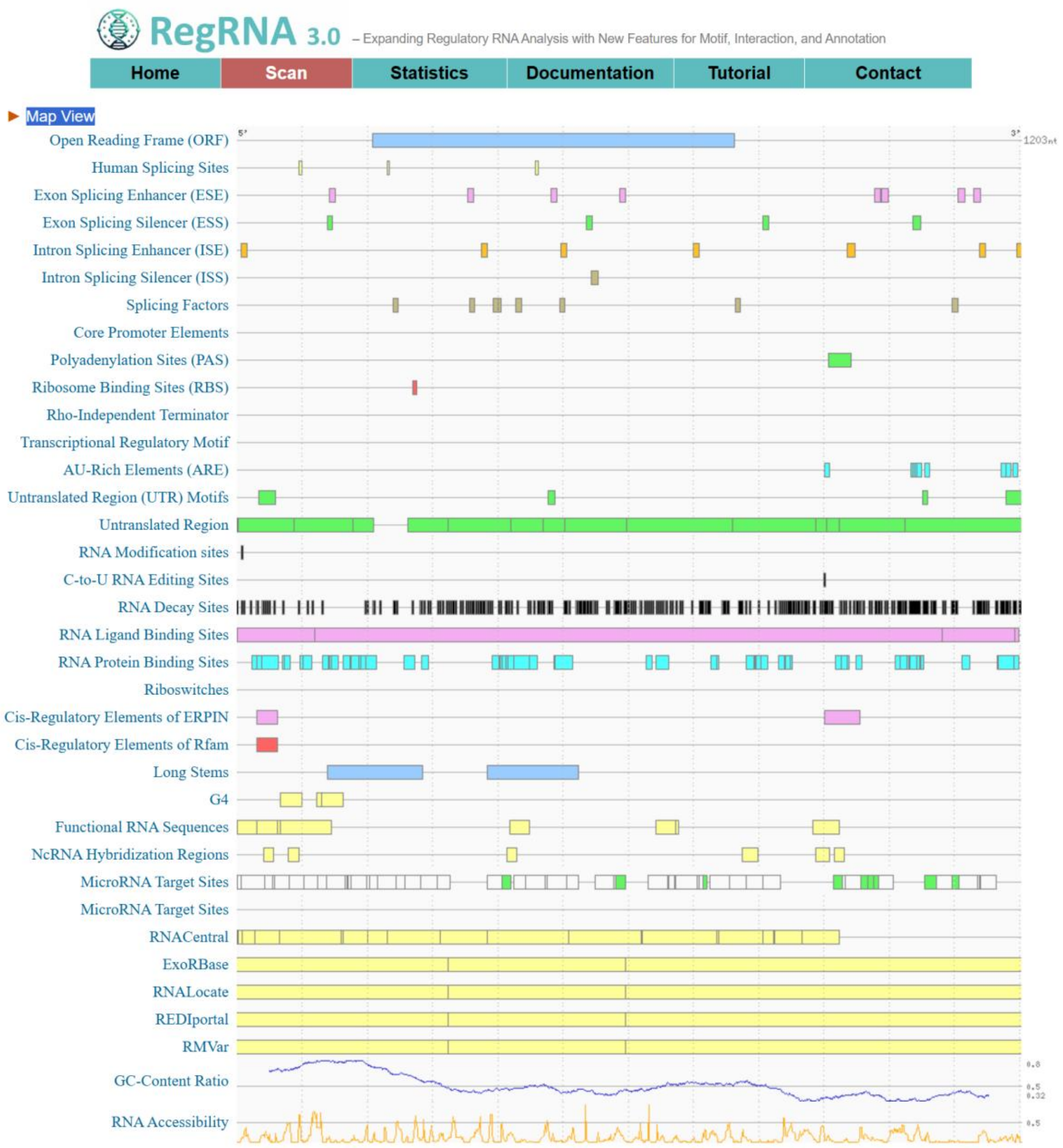

Supplementary Figure 3. *Functional motif scan for the FTH1 mRNA using RegRNA 3.0 identified many various motifs, including IRE, using the Rfam database. All available feature scans were selected, and the detected features can be seen as horizontal tracks which in some cases superimpose due to the independent scanning nature of RegRNA [20].*

***Supplementary Table 1**. External tools integrated in RNAanalyzer[3] with their function, method and our additions to their output.*

Module	Function	External method/tool	Reference	Our contribution
RNA motif scan	Scans for Rfam RNA motifs	Infernal cmscan + Rfam	[8,9]	Integration, parsing, whole-sequence mapping, and structure display
tRNA scan	Scans for tRNAs	tRNAscan-SE	[10]	Integration, parsing, and mapping on the full sequence
miRNA scan	Scans for miRNA regions	nhmmer + miRBase	[13,14]	Integration, parsing, and mapping on the full sequence
RNA binding protein motif scan	Scans for RBP motifs	YAMATK / RBPDB	[11,21]	Integration, parsing, and mapping on the full sequence
miRNA target prediction	Scans for miRNA target sites	Miranda + miRBase, restricted to inferred 3′ UTR when available	[13,15]	Wrapper, parsing, and biologically informed UTR-restricted scanning to reduce runtime and improve relevance
Coding potential	Predicts coding potential / gene annotation	CPC2 / AUGUSTUS	[6,7]	Integration, parsing, transcript-level handling of multiple CDSs, and mapping on the full sequence
Riboswitch scanning	Finds riboswitches	Infernal cmscan + Rfam	[8,9]	Integration, parsing, added RNAfold energy calculations, stem calculations, and annotation
Folding	Predicts RNA secondary structure	RNAfold	[18]	Integration and parsing
Structure visualization	Visualizes predicted RNA structure	VARNA / FORNA	[16,17]	Integration and feature-label annotation

***Supplementary Table 2**. Internal modules of RNAanalyzer[3]. The core methodology and their intended functions are stated.*

Module	Function	Core method	Our contribution	Validation
IRE	Scans for iron-responsive elements	Rule-based motif and structure detection	Updated in-house module incorporating canonical IRE patterns	Targeted internal validation
AU-rich regions	Finds AU-rich regions / ARE-like motifs	Rule-based motif and sequence-content detection	In-house detection module	Targeted internal validation
Trans-splicing	Scans for trans-splicing motifs/features	Rule-based motif and structure detection	In-house detection module	Targeted internal validation
UTR prediction	Infers 5′ and 3′ UTR regions	Hybrid inference from CDS boundaries plus polyA-related motifs/signals, using external CDS prediction as upstream input	Inference layer combining CDS prediction with motif-guided 3′ UTR interpretation	Inferred / predicted from CPC2 or Augustus.
SM / snRNP site scan	Scans for splice-related SM/snRNP recognition motifs	Rule-based motif detection	In-house motif-scanning module	Targeted internal validation
CstF motifs	Scans for CstF-binding GU-rich motifs / downstream sequence elements	Rule-based motif detection	In-house motif-scanning module	Legacy / No validation

***Supplementary Table 3**. Benchmarking of the internal modules. The metrics were calculated using the formula given below.*

Module	Positive-control	Negative-control	Output	TP / TN / FP / FN	Sensitivity	Specificity	Precision
**IRE**	Curated canonical IRE-containing transcripts	Matched loop / bulge / stem mutants; random mRNA seq	Detected IRE motif & structure	19 / 210 / 5 / 0	1.0	0.98	0.79
**AU-rich regions / ARE-like motifs**	Curated ARE-containing transcripts	Random mutations in the detected motif, random mRNA seq	ARE & AU-rich motif detection	10 / 10 / 3 / 0	1.0	0.85	0.77
**Trans-splicing (TRANS2)**	Transcripts containing splice leaders in nematodes	GGUA mutants, Sm-site mutants, stem-disruption mutants, random mRNA seq	Hits including Stem1, Stem2, Stem3, Sm-site-like motif	8 / 17 / 6 / 4	0.67	0.74	0.57
**SM / snRNP site scan**	Curated sets of sm-site containing transcripts	Mutations in the recognized motifs; random mRNA seq	Detected Sm or snRNP-like motif	5 / 6 / 10 / 0	1.0	0.38	0.33

$$Specificity = \frac{TN}{TN+FP} \qquad Sensitivity = \frac{TP}{TP+FN} \qquad Precision = \frac{TP}{TP+FP}$$

where,

TP = True positives, TN = True Negatives, FP = False Positives, FN = False Negatives

Supplementary Table 4. *Structural features offered by RNAanalyzer3.*

Structure-based Feature	RNAanalyzer3 Offers	Biological relevance
RNA secondary structure analysis	Calculates secondary RNA structure and provides visualization.	Provides direct structure analysis relevant for RNA function.
Alternative Folding analysis	Added ability to check for alternative secondary structure analysis.	MFE structure does not always reflect biology, this can help to check what other conformations can exist.
Different Folding routines	Enables prediction of MFE (default), centroid and pseudoknot structures.	Deepens understanding of RNA structure and various structural interpretations.
Structure annotation	Detected features are overlayed on the whole structure of the transcript. Allows zooming and panning to easier visualization.	Helps in interpretations of regulatory and structural relationships.
Integrations of structural context in core modules	Modules like IRE, trans-splice, riboswitch, structural region scan, UTR inference uses predicted structures to add structural context.	Allows direct structural relation to the predicted / detected feature.

Supplementary Table 5. *IREs detected by the updated IRE module of RNAanalyzer[3] as described in [1] and the newly annotated IRE in PFN2 [22].*

Tier	Gene	Common Name	UTR side	Notes
**Canonical / classic**	**FTH1**	Ferritin heavy chain	5′	Classic IRE
**Canonical / classic**	**FTL**	Ferritin light chain	5′	Classic IRE
**Canonical / classic**	**TFRC**	Transferrin receptor 1	3′	Multiple 3′ IREs
**Canonical / classic**	**ALAS2**	Erythroid aminolevulinate synthase	5′	Classic erythroid IRE
**Canonical / classic**	**ACO2**	Mitochondrial aconitase	5′	Well-accepted functional IRE
**Canonical / classic**	**SLC40A1 / FPN1**	Ferroportin	5′	Functional 5′ IRE
**Canonical / classic**	**SLC11A2 / DMT1**	Divalent metal transporter 1	3′	IRE containing isoform
**Canonical / classic**	**EPAS1 / HIF2A**	Hypoxia-inducible factor 2α	5′	Relatively new IRE
**Canonical / classic**	**CDC14A**	Cell division cycle 14A	3′	Isoform-specific functional IRE
**Canonical / classic**	**SDHB**	Succinate dehydrogenase subunit B	5′	Well accepted IRE in Drosophila
**Validated non-canonical / functional IRE-like**	**PFN2**	Profilin 2	3′	Functional, conserved, non-canonical

Supplementary Table 6. *Detailed comparison with other webservers and their conceptual differences and which areas are covered by RNAanalyzer[3].*

Tool	Coding context	UTR context	Regulatory motifs	Interaction features	RNA class	Secondary structure	Alternative structure views	Integrated transcript-level interpretation
**RNAanalyzer3**	**Yes**<br>CPC2, Augustus	**Yes**	**Yes**<br>IRE, Rfam motif scan, Trans-splicing, Au-rich elements, Riboswitches, Sm-sites	**Yes**<br>RBP motifs and miRNA target sites	**Yes**<br>tRNAs, miRNAs, contextual lncRNAs	**Yes**<br>Sequence level visualization, combined with smaller motif level support integrated in many motif scans	**Yes**<br>MFE, centroid, pseudoknot visualization, and suboptimal structures	**Yes**<br>tabulated multi-layer output with annotated interactive structure, coordinates, links, and downloadable graphics
**RegRNA 3.0**	**Limited**<br>ORF annotation	**Partial**<br>UTR motif scans but no UTR inference	**Extensive**<br>17 functional motif/site classes, including splicing, polyadenylation, UTR motifs, AREs, riboswitches, G-quadruplexes, RNA elements, and more	**Broad**<br>miRNA targets, ncRNA hybridization, TRANSFAC motifs, RNA–ligand interactions, and RBPs	**Yes**<br>General ncRNA (RNAcentral), Exosome RNA, RNA subcellular location, Editing events, modification	**Partial**<br>Not whole sequence; limited to recognized motifs	**Limited**<br>2D and 3D structure prediction for recognized motifs	**Yes**<br>A broad integrated annotation platform, but focuses on regulatory motifs
**SIREs 3.0**	**No**	**No**	**Limited / Specialized**<br>Focused on IREs	**No**	**No**	**Specific**<br>structure-aware IRE prediction	**Specific**<br>Limited to IRE	**Limited**<br>Specific for IRE analysis, but not a general transcript interpretation platform
**RNAfold (ViennaRNA Web)**	**No**	**No**	**No**	**No**	**No**	**Yes**<br>Specialized for secondary structure prediction	**Specialized**<br>MFE and centroid structure prediction and visualization	**No**<br>structure-focused rather than multi-layer transcript annotation
**RNAcentral**	**No**	**No**	**Yes**<br>Integrated Rfam search	**No**	**Extensive**<br>broad ncRNA sequence/resource integration	**Limited**<br>includes large-scale 2D structure integration and visualization	**No**	**Limited**<br>Broad noncoding and regulatory RNA class annotation

Supplementary Table 7. *Benchmarking of canonical and functionally validated IRE elements with SIREs webserver showing the tight detectors of RNAanalyzer3 boasts better specificity and precision as compared to SIREs 3.0 using the same internal targeted validation dataset.*

**Method**	**Positive WTs (n)**	**Mutations (n)**	**Random sequences (n)**	**TP**	**FN**	**TN**	**FP**	**Sensitivity**	**Specificity**	**Precision**
RNAanalyzer3	19	30	185	19	0	210	5	1.00	0.98	0.79
SIREs	19	30	185	19	0	133	82	1.00	0.62	0.19

Supplementary Table 8*. Dataset used for IRE targeted validation. The 10 canonical IRE and their mouse counterparts were taken as positive control [1]. For negative controls, 3 types of mutations and random negative mRNA transcripts were taken.*

Control	Gene / IRE	Accession	Organism	Detection	Single loop mutants detected	Double loop mutants detected	Bulge mutants detected
Positive	SDHB	NM_057753.5	Drosophila melanogaster	Detected	No	No	No
Positive	ALAS2	NM_000032.5	Homo sapiens	Detected	Yes	No	No
Positive	FTL	NM_000146.4	Homo sapiens	Detected	No	No	No
Positive	SLC11A2	NM_000617.3	Homo sapiens	Detected	No	No	No
Positive	ACO2	NM_001098.3	Homo sapiens	Detected	No	No	No
Positive	EPAS1	NM_001430.5	Homo sapiens	Detected	No	No	No
Positive	FTH1	NM_002032.3	Homo sapiens	Detected	No	No	No
Positive	TFRC	NM_003234.4	Homo sapiens	Detected	No	No	No
Positive	CDC14A	NM_003672.4	Homo sapiens	Detected	No	No	No
Positive	SLC40A1	NM_014585.6	Homo sapiens	Detected	No	No	No
Positive	PFN2	NM_053024.4	Homo sapiens	Detected	-	-	-
Positive	SLC11A2	NM_001356952.1	Mus musculus	Detected	-	-	-
Positive	ALAS2	NM_009653.4	Mus musculus	Detected	-	-	-
Positive	FTH1	NM_010239.2	Mus musculus	Detected	-	-	-
Positive	FTL1	NM_010240.2	Mus musculus	Detected	-	-	-
Positive	TFRC	NM_011638.4	Mus musculus	Detected	-	-	-
Positive	SLC40A1	NM_016917.2	Mus musculus	Detected	-	-	-
Positive	PFN2	NM_019410.3	Mus musculus	Detected	-	-	-
Positive	ACO2	NM_080633.2	Mus musculus	Detected	-	-	-
Negative	ADCY3	NM_001320613.2	Homo sapiens	No	-	-	-
Negative	AFMID	NM_001010982.5	Homo sapiens	No	-	-	-
Negative	AFTPH	NM_001002243.3	Homo sapiens	No	-	-	-
Negative	ALMS1-IT1	NR_046762.1	Homo sapiens	No	-	-	-
Negative	ALPL	NM_000478.6	Homo sapiens	No	-	-	-
Negative	AMT	NM_000481.4	Homo sapiens	No	-	-	-
Negative	ANGPTL2	NM_012098.3	Homo sapiens	No	-	-	-
Negative	ANKRD11	NM_001256182.2	Homo sapiens	No	-	-	-
Negative	ANKRD2	NM_001129981.3	Homo sapiens	No	-	-	-
Negative	ARHGEF35	NM_001003702.3	Homo sapiens	No	-	-	-
Negative	ATP6V0C	NM_001198569.2	Homo sapiens	No	-	-	-
Negative	ATP5IF1	NM_016311.5	Homo sapiens	No	-	-	-
Negative	BOLL	NM_001284358.2	Homo sapiens	No	-	-	-
Negative	C10ORF105	NM_001164375.3	Homo sapiens	No	-	-	-
Negative	HIKESHI	NM_001322404.2	Homo sapiens	No	-	-	-
Negative	C19ORF38	NM_001136482.3	Homo sapiens	No	-	-	-
Negative	LINC02871	NR_146878.1	Homo sapiens	No	-	-	-
Negative	LINC03040	NR_160954.1	Homo sapiens	No	-	-	-
Negative	CFAP95	NM_001010940.3	Homo sapiens	No	-	-	-
Negative	CAB39	NM_001130849.2	Homo sapiens	No	-	-	-
Negative	CACNA1C	NM_000719.7	Homo sapiens	No	-	-	-
Negative	CAMK2A	NM_001363989.1	Homo sapiens	No	-	-	-
Negative	CASC23	NR_125366.1	Homo sapiens	No	-	-	-

Negative	CATSPER4	NM_198137.2	Homo sapiens	No	-	-	-
Negative	CBX2	NM_005189.3	Homo sapiens	No	-	-	-
Negative	CCDC30	NM_001080850.4	Homo sapiens	No	-	-	-
Negative	CDC6	NM_001254.4	Homo sapiens	No	-	-	-
Negative	CDCA7L	NM_001127370.3	Homo sapiens	No	-	-	-
Negative	CEP57L1	NM_001083535.3	Homo sapiens	Detected	-	-	-
Negative	CHD1L	NM_001256336.3	Homo sapiens	No	-	-	-
Negative	CHST2	NM_004267.5	Homo sapiens	No	-	-	-
Negative	CLECL1	NM_001441804.1	Homo sapiens	No	-	-	-
Negative	CLMN	NM_024734.4	Homo sapiens	No	-	-	-
Negative	CMTM6	NM_017801.3	Homo sapiens	No	-	-	-
Negative	COX7B2	NM_130902.3	Homo sapiens	No	-	-	-
Negative	CYP51A1	NM_000786.4	Homo sapiens	No	-	-	-
Negative	DALIR	NR_197589.1	Homo sapiens	No	-	-	-
Negative	DIAPH3	NM_001042517.2	Homo sapiens	Detected	-	-	-
Negative	DNAI2	NM_001172810.3	Homo sapiens	No	-	-	-
Negative	EFNA3	NM_004952.5	Homo sapiens	No	-	-	-
Negative	ELAC2	NM_001165962.2	Homo sapiens	No	-	-	-
Negative	EPB41L4A	NM_001347887.2	Homo sapiens	No	-	-	-
Negative	ESM1	NM_001135604.2	Homo sapiens	No	-	-	-
Negative	EEIG2	NM_001010883.3	Homo sapiens	No	-	-	-
Negative	FAM185A	NM_001145268.2	Homo sapiens	No	-	-	-
Negative	FAM216B	NM_001318932.2	Homo sapiens	No	-	-	-
Negative	FAM86B2	NM_001137610.3	Homo sapiens	No	-	-	-
Negative	FAM95A	NR_038409.1	Homo sapiens	No	-	-	-
Negative	FBF1	NM_001319193.2	Homo sapiens	No	-	-	-
Negative	FOXC2-AS1	NR_125795.1	Homo sapiens	No	-	-	-
Negative	FTCD	NM_001320412.2	Homo sapiens	No	-	-	-
Negative	GCLC	NM_001197115.2	Homo sapiens	No	-	-	-
Negative	GCSAM	NM_001190259.2	Homo sapiens	No	-	-	-
Negative	GHR	NM_000163.5	Homo sapiens	No	-	-	-
Negative	GLRA1	NM_000171.4	Homo sapiens	No	-	-	-
Negative	GSTA5	NM_153699.3	Homo sapiens	No	-	-	-
Negative	GTSF1	NM_144594.3	Homo sapiens	No	-	-	-
Negative	GUCY2C	NM_004963.4	Homo sapiens	No	-	-	-
Negative	HECW1-IT1	NR_135295.1	Homo sapiens	No	-	-	-
Negative	HEIH	NR_045680.1	Homo sapiens	No	-	-	-
Negative	HIGD2A	NM_138820.4	Homo sapiens	No	-	-	-
Negative	HIGD2B	NM_001350932.3	Homo sapiens	No	-	-	-
Negative	H1-3	NM_005320.3	Homo sapiens	No	-	-	-
Negative	H4C7	NM_003547.3	Homo sapiens	No	-	-	-
Negative	HMMR	NM_001142556.2	Homo sapiens	No	-	-	-
Negative	HOXB9	NM_024017.5	Homo sapiens	No	-	-	-
Negative	HSCB	NM_001318314.2	Homo sapiens	No	-	-	-
Negative	IDH1-AS1	NR_046452.1	Homo sapiens	No	-	-	-
Negative	IDO2	NM_001395206.1	Homo sapiens	No	-	-	-
Negative	INTS2	NM_001330417.2	Homo sapiens	No	-	-	-

Negative	INTS3	NM_001324475.2	Homo sapiens	No	-	-	-
Negative	ITGB7	NM_000889.3	Homo sapiens	No	-	-	-
Negative	JAK1	NM_001320923.2	Homo sapiens	No	-	-	-
Negative	KANSL1L	NM_001307976.2	Homo sapiens	No	-	-	-
Negative	KAZALD1	NM_001319303.2	Homo sapiens	No	-	-	-
Negative	KDELR1	NM_006801.3	Homo sapiens	No	-	-	-
Negative	LACTBL1	NM_001289974.2	Homo sapiens	No	-	-	-
Negative	LGALS1	NM_002305.4	Homo sapiens	No	-	-	-
Negative	LHX5-AS1	NR_126425.1	Homo sapiens	No	-	-	-
Negative	LHX6	NM_001242333.2	Homo sapiens	No	-	-	-
Negative	LILRB2	NM_001080978.4	Homo sapiens	No	-	-	-
Negative	LINC00926	NR_024433.1	Homo sapiens	No	-	-	-
Negative	LINC01152	NR_110124.1	Homo sapiens	No	-	-	-
Negative	LINC01208	NR_109968.1	Homo sapiens	No	-	-	-
Negative	LINC01219	NR_126400.1	Homo sapiens	No	-	-	-
Negative	LINC01582	NR_120325.1	Homo sapiens	No	-	-	-
Negative	LINC01601	NR_131768.1	Homo sapiens	No	-	-	-
Negative	LIPE-AS1	NR_073179.1	Homo sapiens	No	-	-	-
Negative	LRTM1	NM_001304389.2	Homo sapiens	No	-	-	-
Negative	LY6D	NM_003695.3	Homo sapiens	No	-	-	-
Negative	MDH1B	NM_001039845.3	Homo sapiens	No	-	-	-
Negative	MNT	NM_020310.3	Homo sapiens	No	-	-	-
Negative	MYO16-AS1	NR_047700.1	Homo sapiens	No	-	-	-
Negative	NELL2	NM_001145107.2	Homo sapiens	No	-	-	-
Negative	NHSL1	NM_001144060.2	Homo sapiens	No	-	-	-
Negative	MYO1C	NM_001080779.2	Homo sapiens	No	-	-	-
Negative	NPPA	NM_006172.4	Homo sapiens	No	-	-	-
Negative	OCLN	NM_001205254.2	Homo sapiens	No	-	-	-
Negative	OR10H1	NM_013940.4	Homo sapiens	No	-	-	-
Negative	OR6P1	NM_001160325.2	Homo sapiens	No	-	-	-
Negative	P2RX2	NM_001282164.2	Homo sapiens	No	-	-	-
Negative	PATE3	NM_001129883.4	Homo sapiens	No	-	-	-
Negative	PAX8-AS1	NR_015377.2	Homo sapiens	No	-	-	-
Negative	PCDHGA5	NM_018918.3	Homo sapiens	No	-	-	-
Negative	D-ASPARTATE	NM_001286782.1	Homo sapiens	No	-	-	-
Negative	PDE8B	NM_001029851.4	Homo sapiens	No	-	-	-
Negative	PEF1	NM_001359651.2	Homo sapiens	No	-	-	-
Negative	PGR	NM_000926.4	Homo sapiens	No	-	-	-
Negative	PHF12	NM_001033561.2	Homo sapiens	No	-	-	-
Negative	PI4KA	NM_001362862.2	Homo sapiens	No	-	-	-
Negative	INACTIVE	NM_001243079.2	Homo sapiens	No	-	-	-
Negative	PLXNA1	NM_032242.4	Homo sapiens	No	-	-	-
Negative	PNKD	NM_001077399.3	Homo sapiens	No	-	-	-
Negative	POGLUT1	NM_152305.3	Homo sapiens	No	-	-	-
Negative	POLD1	NM_001256849.1	Homo sapiens	No	-	-	-
Negative	POLR3G	NM_001370351.1	Homo sapiens	No	-	-	-
Negative	POTEJ	NM_001277083.2	Homo sapiens	No	-	-	-

Negative	PPP2R1B	NM_001177562.2	Homo sapiens	No	-	-	-
Negative	PRAC2	NM_001282275.2	Homo sapiens	No	-	-	-
Negative	PTAFR	NM_000952.5	Homo sapiens	No	-	-	-
Negative	PTGES3	NM_001282601.2	Homo sapiens	No	-	-	-
Negative	PXN	NM_001080855.3	Homo sapiens	No	-	-	-
Negative	RC3H1	NM_001300850.1	Homo sapiens	No	-	-	-
Negative	RIMS3	NM_014747.3	Homo sapiens	No	-	-	-
Negative	RNF208	NM_001388297.1	Homo sapiens	No	-	-	-
Negative	RNU5F-1	NR_002753.8	Homo sapiens	No	-	-	-
Negative	ROPN1	NM_001317774.2	Homo sapiens	No	-	-	-
Negative	RPS19BP1	NM_194326.4	Homo sapiens	No	-	-	-
Negative	RPS6KA1	NM_001006665.2	Homo sapiens	No	-	-	-
Negative	SCARA3	NM_016240.3	Homo sapiens	No	-	-	-
Negative	SCARNA21B	NR_135613.1	Homo sapiens	No	-	-	-
Negative	PUTATIVE	NM_016002.3	Homo sapiens	No	-	-	-
Negative	SCN1A	NM_001165963.4	Homo sapiens	No	-	-	-
Negative	SEC14L5	NM_014692.2	Homo sapiens	No	-	-	-
Negative	SEMA6D	NM_001198999.2	Homo sapiens	No	-	-	-
Negative	O-PHOSPHOSERINE	NM_001410714.1	Homo sapiens	No	-	-	-
Negative	SLC33A1	NM_001190992.2	Homo sapiens	No	-	-	-
Negative	SLC35E2B	NM_001110781.3	Homo sapiens	No	-	-	-
Negative	SLC35F2	NM_017515.5	Homo sapiens	No	-	-	-
Negative	SLC7A9	NM_001126335.2	Homo sapiens	No	-	-	-
Negative	SLFN11	NM_001104587.2	Homo sapiens	No	-	-	-
Negative	SMG8	NM_018149.7	Homo sapiens	No	-	-	-
Negative	ILF3	NR_024221.1	Homo sapiens	No	-	-	-
Negative	SNORA21	NR_002576.1	Homo sapiens	No	-	-	-
Negative	SNORA80E	NR_002974.1	Homo sapiens	No	-	-	-
Negative	SNORD114-20	NR_003213.1	Homo sapiens	No	-	-	-
Negative	SNORD131	NR_132974.1	Homo sapiens	No	-	-	-
Negative	SNORD15A	NR_000005.1	Homo sapiens	No	-	-	-
Negative	SON	NM_001291411.2	Homo sapiens	No	-	-	-
Negative	SPATA31A6	NM_001145196.1	Homo sapiens	No	-	-	-
Negative	SRGAP2-AS1	NR_104189.1	Homo sapiens	No	-	-	-
Negative	SSTR5-AS1	NR_027242.1	Homo sapiens	No	-	-	-
Negative	ST8SIA2	NM_001330416.2	Homo sapiens	No	-	-	-
Negative	STX12	NM_177424.3	Homo sapiens	No	-	-	-
Negative	MAP3K7	NM_006116.3	Homo sapiens	No	-	-	-
Negative	TDRD10	NM_001098475.2	Homo sapiens	No	-	-	-
Negative	TET1	NM_001406365.1	Homo sapiens	No	-	-	-
Negative	SPMAP2	NM_016585.5	Homo sapiens	No	-	-	-
Negative	THOC1	NM_005131.3	Homo sapiens	No	-	-	-
Negative	THSD4-AS2	NR_120346.1	Homo sapiens	No	-	-	-
Negative	THSD4	NM_001286429.2	Homo sapiens	No	-	-	-
Negative	TMEM147	NM_001242597.2	Homo sapiens	No	-	-	-
Negative	TMEM181	NM_001376817.1	Homo sapiens	Detected	-	-	-
Negative	TMEM26-AS1	NR_120643.1	Homo sapiens	No	-	-	-

Negative	TMEM44	NM_001011655.3	Homo sapiens	No	-	-	-
Negative	RXYLT1-AS1	NR_126167.1	Homo sapiens	No	-	-	-
Negative	TRPM4	NM_001195227.2	Homo sapiens	No	-	-	-
Negative	TSC22D1	NM_001243797.2	Homo sapiens	No	-	-	-
Negative	GFUS	NM_001317783.2	Homo sapiens	No	-	-	-
Negative	TXNDC12-AS1	NR_126385.1	Homo sapiens	No	-	-	-
Negative	UBA6-DT	NR_015439.1	Homo sapiens	No	-	-	-
Negative	UBE2Q2P16	NR_166151.1	Homo sapiens	No	-	-	-
Negative	UGT2A2	NM_001105677.2	Homo sapiens	No	-	-	-
Negative	UGT2B17	NM_001077.4	Homo sapiens	No	-	-	-
Negative	SELENOS	NM_018445.6	Homo sapiens	No	-	-	-
Negative	XKR7	NM_001011718.2	Homo sapiens	No	-	-	-
Negative	ZDHHC16	NM_001287803.2	Homo sapiens	No	-	-	-
Negative	ZNF106	NM_001284306.2	Homo sapiens	No	-	-	-
Negative	ZNF446	NM_001304453.1	Homo sapiens	No	-	-	-
Negative	ZNF487	NM_001355444.3	Homo sapiens	No	-	-	-
Negative	ZNF548	NM_001172773.2	Homo sapiens	No	-	-	-
Negative	ZNF675	NM_138330.3	Homo sapiens	No	-	-	-
Negative	ZNF732	NM_001137608.3	Homo sapiens	No	-	-	-
Negative	ZNF785	NM_152458.7	Homo sapiens	Detected	-	-	-
Negative	ZNRF3-AS1	NR_046851.1	Homo sapiens	No	-	-	-

***Supplementary Table 9**. Dataset used for ARE targeted validation.*[1]

ID	Group	Gene / Source	Accession	Region	Observed hit in Exprected Region	No. of hits	Start	End	Representative Seq	Mis-matches	Score	Quality
ARE_POS_01	Positive	TNF	NM_000594.4	3' UTR	Yes	4	1341	1356	Uuauuuauuauuuauu	0	10,	Good.
ARE_POS_02	Positive	CSF2	NM_000758.4	3' UTR	Yes	3	700	716	uuauuuauuuauuuauu	0	10	Good,
ARE_POS_03	Positive	FOS	NM_005252.4	3' UTR;	Yes	2	1940	1953	Aauuuauuuauuaa	0	5,	Moderate
ARE_POS_04	Positive	MYC	NM_002467.6	3' UTR	Yes	1	3405	3413	auauuuauu	0	6.0	Moderate
ARE_POS_05	Positive	IL3	NM_000588.4	3' UTR	Yes	3	756	770	uauuuauuuauuuau	0	10	Good
ARE_POS_06	Positive	IL2	NM_000586.4	3' UTR	Yes	3	785	799	Uauuuauuuaaauau	0	7,	Good
ARE_POS_07	Positive	PTGS2	NM_000963.4	3' UTR	Yes	6	1962	1976	Uauuuauuuauuuau	0	10	Good
ARE_POS_08	Positive	VEGFA	NM_003376.6	~3' UTR	Yes	3	2914	2924	Uuauuuaauau	0	7,	Good
ARE_POS_09	Positive	JUN	NM_002228.4	3' UTR	Yes	1	3209	3219	aauauuuauaa	0	5	Moderate
ARE_POS_10	Positive	IL6	NM_000600.5	3' UTR	Yes	4	828	844	Uaauuauuuuuaauuua	0	6	Moderate,
ARE_MUT_01	Mutant negative	TNF_mut	-	-	No	-	-	-	-	-	-	-
ARE_MUT_02	Mutant negative	CSF2_mut	-	-	No	-	-	-	-	-	-	-
ARE_MUT_03	Mutant negative	FOS_mut	-	-	No	-	-	-	-	-	-	-
ARE_MUT_04	Mutant negative	MYC_mut	-	-	No	-	-	-	-	-	-	-
ARE_MUT_05	Mutant negative	IL3_mut	-	-	No	-	-	-	-	-	-	-
ARE_MUT_06	Mutant negative	IL2_mut	-	-	No	-	-	-	-	-	-	-
ARE_MUT_07	Mutant negative	PTGS2_mut	-	-	Yes	6	1969	1978	Uuauuuauau	0	7	Good
ARE_MUT_08	Mutant negative	VEGFA_mut	-	-	No	-	-	-	-	-	-	-
ARE_MUT_09	Mutant negative	JUN_mut	-	-	No	-	-	-	-	-	-	-
ARE_MUT_10	Mutant negative	IL6_mut	-	-	No	.	-	-	-	-	-	-

ARE_NEG_01	Background negative	ACTB	NM_001101.5	-	Yes	1	1320	1335	Uuuuuuuuuuuuuuuu	0	4	Moderate
ARE_NEG_02	Background negative	TUBB	NM_178014.4	-	No	-	-	-	-	-	-	-
ARE_NEG_03	Background negative	RPLP0	NM_001002.4	-	No	-	-	-	-	-	-	-
ARE_NEG_04	Background negative	HPRT1	NM_000194.3	-	No	-	-	-	-	-	-	-
ARE_NEG_05	Background negative	SDHA	NM_004168.4	-	No	-	-	-	-	-	-	-
ARE_NEG_06	Background negative	PPIA	NM_021130.5	-	Yes	1	1293	1310	uuuuuuuuuuuuuuuuuu	0	4	Moderate
ARE_NEG_07	Background negative	GAPDH	NM_002046.7	-	No	-	-	-	-	-	-	-
ARE_NEG_08	Background negative	RPL13A	NM_012423.4	-	No	-	-	-	-	-	-	-
ARE_NEG_09	Background negative	B2M	NM_004048.4	-	No	-	-	-	-	-	-	-
ARE_NEG_10	Background negative	EEF1A1	NM_001402.6	-	No	-	-	-	-	-	-	-

[1]The positive controls were correctly detected by the module in their described regions with two backgrounds random negative transcripts detected as false positives due to U-rich sequence recognition. The module also showed high resistance to mutant controls with one being detected as false positive.

Supplementary Table 10. *Dataset used for TRANS-Splicing module targeted validation.* [2]

Name	Accession	Control	Detection	Detected upsteam region (Stem 1)	Detected Motif (Stem2)	Detected Support (Stem3)	Sm-site
Leptomonas collosoma small spliced leader	K02633.1	+ve	Yes	GAACAGUUUCUGUACUUCAUUGGUAUGUAGAGACUUCCA	CCAGAACCUAGUUCUGA	ACCGAGCUUUCGGGCUUUUUUUUUACUUUUUCGGGUAC	AAAUUUUGG
Trypanosoma cruzi small spliced leader	K02631.1	+ve	No	-	-	-	-
T.brucei small spliced leader	K02629.1	+ve	No		-		
Trypanosomatidae sp. 5VL-B trans-spliced leader	DQ864307.1	+ve	Yes	UUACAGUUUCUGUACUUUAUUGGUAUGAGAAACUUCCAG	GAAACUUCCAGAACUUUACUAGUUCUAGGUA	AACAAAAGCCUUCGGGCUUUUUUUUUAUAUUUUUUUUUACA	AAAUUUUGG
Blastocrithidia raabei isolate Cor9 clone 1 trans-spliced leader	MN380295.1	+ve	Yes	GUUACAGUUUCUGUACUUAUUGGUAUGAGAAACUUCCAG	AGAAACUUCCAGAACUUUCAUAGUUCUAGGGA	AACAAAAGCCUUCGGGCUUUUUUUUUAUUUUUUUUUUUACG	AAAUUUUGG
A.lumbricoides spliced leader	M27961.1	+ve	Yes	GGUUUAAUUACCCAAGUUUGAGGUAAUUCCGUGUUUCAG	UCCGUGUUUCAGCUCAGUGCUUCUAUCGGCUCUGUGGCUUGAAAAUA	AACGCUUUGCCGUAUGGCGAAGCACUUUUGAAACGUUUAAA	AAAUUUUGG
C.elegans actin spliced leader	M17403.1	+ve	Yes	GGUUUAAUUACCCAAGUUUGAGGUAAACAUUGAAACUGA	CAUUGAAACUGACCCAAAGAAAUUUGCGUUAGCUAUA	AACGUCUCCUCUCGGGGAGACAAAAAUACUAAGAAAUUCGA	AAAUUUUGG
S.mansoni spliced leader RNA gene	M34074.1	+ve	Yes	AACCGUCACGGUUUUACUCUUGUGAUUUGUUGCAUGGUAAGAACCGUCGACCAAGAAUCGA	-	-	AGUUUUCUUUGG
Wuchereria bancrofti trans-spliced leader[3]	AF297119.1	+ve	Yes	GGUUUAAUUACCCAAGUUUGAGGUAAUUGAAUGUUUCGG	UGAAUGUUUCGGCCCAGAGUUUAGGCUACUGUGGCUUGAAGUAA	AACGUCCUGCAUGUUUGUGGGACAAUAAAUGUGUUUGAAAU	AAAUUUUGG
Trichinella spiralis spliced leader	EF681123.1	+ve	No	-	-	-	-
Ascaris suum spliced leader	AY089966.1	+ve	No	-	-	-	-
Onchocerca volvulus 5S ribosomal RNA	U09024.1	+ve	Yes	GGUUUAAUUACCCAAGUUUGAGGUAAUUGAAUGUUUCUG	UGAAUGUUUCUGCCCAGAGUUUCGACUGCUGUGGCUUGAAGCGA	AACGUCCUGCAUGAGCGGGACAACAAAAAAUGAAAUAUUGG	AAAUUUUGG
Caenorhabditis elegans Myosin-4	NM_061195.7	-ve	No		-		
Caenorhabditis elegans daf-2	NM_065249.7	-ve	No		-		
Caenorhabditis elegans egl-1	NM_074174.6	-ve	No		-		
Caenorhabditis elegans cdc-42	NM_063197.10	-ve	No		-		
Homo sapiens insulin	NM_000207.3	-ve	No		-		
Homo sapiens somatostatin	NM_001048.4	-ve	No		-		

Homo sapiens beta-2-microglobulin	NM_004048.4	-ve	No		-		
Homo sapiens hemoglobin subunit alpha 1	NM_000558.5	-ve	No		-		
Homo sapiens C-C motif chemokine ligand 5	NM_002985.3	-ve	No		-		
Homo sapiens TIMP metallopeptidase inhibitor 1	NM_003254.3	-ve	No		-		
Homo sapiens apolipoprotein C3	NM_000040.3	-ve	No		-		
Homo sapiens glyceraldehyde-3-phosphate dehydrogenase	NM_002046.7	-ve	Yes	GUCACCAGGGCUGCUUUUAACUCUGGUAAAGUGGAUAUUGUU	GUGGAUAUUGUUGCCAUCAAUGACCCCUUCAUUGACCUCAACUACAUG	UGUUCCAAUAUGAUUCCACCCAUGGCAAAUUCCAUGGCACC	GGUUUACA
Homo sapiens cyclin dependent kinase inhibitor 1A	NM_000389.5	-ve	No		-		
Leptomonas collosoma small spliced leader	K02633.1	Mut. 3 BP Stem1	Yes	GAACAGUUUCUAUGCUUCAUUGGUAUGUAGAGACUUCCA	CCAGAACCUAGUUCUGA	ACCGAGCUUUCGGGCUUUUUUUUUACUUUUUCGGGUAC	AAAUUUUGG
Trypanosomatidae sp. 5VL-B trans-spliced leader	DQ864307.1	Mut. 1 BP GGUA	No	-	-	-	-
Blastocrithidia raabei isolate Cor9 clone 1 trans-spliced leader	MN380295.1	Mut. 1bp GGUA 1bp stem2	No	-	-	-	-
A.lumbricoides spliced leader	M27961.1	Mut. 4bp stem2	Yes	GGUUUAAUUACCCAAGUUUGAGGUAAUUUUGUGUUUCAG	UUUGUGUUUCAGCUCAGAGCUUCUAUCCGCUCUGUGGCUUGAAAAUA	AACGCUUUGCCGUAUGGCGAAGCACUUUUGAAACGUUUAAA	AAAUUUUGG
C.elegans actin spliced leader	M17403.1	Mut. 2bp stem3	Yes	GGUUUAAUUACCCAAGUUUGAGGUAAACAUUGAAACUGA	CAUUGAAACUGACCCAAAGAAAUUUGCGUUAGCUAUA	AACGUCUCCUCUCGGGGAGACAAAAAUACAAAGAAAUUCGA	AAAUUUUGG
Wuchereria bancrofti trans-spliced leader	AF297119.1	Mut. 2bp sm-site	No	-	-	-	-
Onchocerca volvulus 5S ribosomal RNA	U09024.1	Mut. 3bp sm-site	No	-	-	-	-
Leptomonas collosoma small spliced leader	K02633.1	Mut. 4bp stem1	Yes	AAGAACAGUUUCUGUACUUUGACGGUAUGUAGAGACUUCCA	CUUCCAGAACCUAGUUCUGA	ACCGAGCUUUCGGGCUUUUUUUUUACUUUUUCGGGUAC	AAAUUUUGG

Trypanosomatidae sp. 5VL-B trans-spliced leader	DQ864307.1	Mutation 4bp stem1	Yes	UUACAGUUUCUGUACUUUAUUGGUAUGAGGCACUUCUUG	GGCACUUCUUGAACUUUACUAGUUCUAGGUA	AACAAAAGCCUUCGGGCUUUUUUUUUAUAUUUUUUUUUACA	AAAUUUUGG
S.mansoni spliced leader RNA gene	M34074.1	Mut. 5bp stem1	No	-	-	-	-

[2]Performance was notably better on unrelated background negatives than on matched mutated negatives, indicating that the detector captures parts of the intended motif architecture but is less robust to subtle local perturbations.

[3]Wuchereria bancrofti trans-spliced sequence contains 3 splice motifs, first hit as representation. [23]

Supplementary Table 11. *Dataset used for Sm-site targeted validation.*[4]

Class	RNA / Source	Accession	Reason for inclusion	Detection	Detected Sequence
Positive	U1 snRNA	NR_004430.4	Canonical Sm-site-containing snRNA	Yes	aauuugugg
Positive	U2 snRNA	NR_002716.3	Canonical Sm-site-containing snRNA	Yes	gauuuuugg
Positive	U4 snRNA	NR_003925.2	Canonical Sm-site-containing snRNA	Yes	aauuuuuga
Positive	U5 snRNA	NR_002756.2	Canonical Sm-site-containing snRNA	Yes	Agucuuaa / aauuuuuuga
Positive	U11	NR_004407.1	Extra biological replicate/example	Yes	aauuuuuugg
Mutated negative	U1-derived mutant	derived from positive	2 bp Random mutation	No	-
Mutated negative	U2-derived mutant	derived from positive	1 Bp random mutation	Yes	gguuuuugg
Mutated negative	U4-derived mutant	derived from positive	2 bp random mutation	No	-
Mutated negative	U5-derived mutant motif 1	derived from positive	2 bp random mutation	No	-
Mutated negative	U5-derived mutant motif 2	derived from positive	2 bp random mutation	No	-
Mutated negative	U11-derived mutant	derived from positive	3 bp random mutation	Yes	gguauuugg
Negative	ISN	NM_000207.3	Random	No	-
Negative	SST	NM_001048.4	Random	Yes	Aauuaugg / aauuauga
Negative	B2M	NM_004048.4	Random	Yes	Aauauuga / gguuauaa/ aauguuaa / gauuauugg / gguugugg
Negative	HBA1	NM_000558.5	Random	Yes	ggucuuuga
Negative	CCL5	NM_002985.3	Random	Yes	Aauuguuuga / gguucuga
Negative	TIMP1	NM_003254.3	Random	Yes	aguuuugugg
Negative	CDKN1A	NM_000389.5	Random	Yes	Gguuauga / aguguuga / guauugg / gauucuuag
Negative	TNF	NM_000594.4	Random	Yes	gguucugg
Negative	APOC3	NM_000040.3	Random	Yes	aguucugg
Negative	GAPDH	NM_002046.7	Random	No	-

[4]The module was able to recognize canonical positive controls known for containing Sm-sites, but the module showed over identification in random mutants of the known sites as well as random human transcripts. This can be explained by the small length of the motif and the recognition rule. However, the module is still helpful due to its high sensitivity to the smaller motif pattern.

Supplementary Table 12. *Au-rich regions and RNA binding protein motifs predicted by RNAanalyzer[3] in the TNF mRNA transcript.*

Feature class	Predicted factor / type	Start	End	Sequence	Score / quality / p-value	Region context
ARE	AU-rich region	1341	1356	uuauuuauuauuuauu	10.00, Good	3' UTR
ARE	AU-rich region	1359	1373	uuauuuauuuauuua	9.00, Good	3' UTR
ARE	AU-rich region	1363	1373	uuauuuauuua	6.00, Moderate	3' UTR
ARE	AU-rich region	1384	1392	uauuuauuu	7.00, Good	3' UTR
ARE	AU-rich region	1532	1541	aaauauuuau	6.00, Moderate	3' UTR
RBP motif	SFRS2	25	33	agcagagga	12.084; p = 3.05175781e-05	5' UTR
RBP motif	SFRS1	315	322	gcucuucu	11.373; p = 7.62939453e-05	CDS
RBP motif	SFRS2	417	425	uucucgaac	12.632; p = 3.43322754e-05	CDS
RBP motif	SFRS2	417	426	uucucgaacc	12.994; p = 2.19345093e-05	CDS
RBP motif	SNRPA	1329	1336	uuugcacu	12.479; p = 6.10351562e-05	3' UTR
RBP motif	ZFP36	1342	1350	uuauuuauu	15.585; p = 3.81469727e-06	3' UTR
RBP motif	ELAVL2	1348	1361	auuauuuauuuauu	13.027; p = 1.54711306e-05	3' UTR
RBP motif	ZFP36	1351	1361	auuuauuuauu	6.635; p = 3.14712524e-05	3' UTR
RBP motif	PUM	1363	1378	uuuauuuauuuacaga	19.213; p = 7.93952495e-08	3' UTR
RBP motif	KHDRBS3	1526	1541	uuuuuuaaaauauuua	14.516; p = 7.07292929e-06	3' UTR
RBP motif	NCL	1635	1645	ccuacuauuca	13.443; p = 1.00135803e-05	3' UTR

References

1. Volz, K. (2021) Conservation in the Iron Responsive Element Family. *Genes*, **12**, 1365.

http://www.ncbi.nlm.nih.gov/pubmed/doi:10.3390/genes12091365

2. Henderson, B.R., Menotti, E. and Kühn, L.C. (1996) Iron regulatory proteins 1 and 2 bind distinct sets of RNA target sequences. *J Biol Chem*, **271**, 4900-4908.

http://www.ncbi.nlm.nih.gov/pubmed/8617762

http://dx.doi.org/10.1074/jbc.271.9.4900

3. Bengert, P. and Dandekar, T. (2003) A software tool-box for analysis of regulatory RNA elements. *Nucleic Acids Res*, **31**, 3441-3445.

http://www.ncbi.nlm.nih.gov/pubmed/12824342

http://dx.doi.org/10.1093/nar/gkg568

4. Dandekar, T., Stripecke, R., Gray, N.K., Goossen, B., Constable, A., Johansson, H.E. and Hentze, M.W. (1991) Identification of a novel iron-responsive element in murine and human erythroid delta-aminolevulinic acid synthase mRNA. *Embo j*, **10**, 1903-1909.

http://www.ncbi.nlm.nih.gov/pubmed/2050126

http://dx.doi.org/10.1002/j.1460-2075.1991.tb07716.x

5. Dandekar, T. and Sibbald, P.R. (1990) Trans-splicing of pre-MRNA is predicted to occur in a wide range of organisms including vertebrates. *Nucleic Acids Research*, **18**, 4719-4725.

http://dx.doi.org/10.1093/nar/18.16.4719

6. Kang, Y.-J., Yang, D.-C., Kong, L., Hou, M., Meng, Y.-Q., Wei, L. and Gao, G. (2017) CPC2: a fast and accurate coding potential calculator based on sequence intrinsic features. *Nucleic Acids Research*, **45**, W12-W16.

http://dx.doi.org/10.1093/nar/gkx428

7. Stanke, M., Diekhans, M., Baertsch, R. and Haussler, D. (2008) Using native and syntenically mapped cDNA alignments to improve de novo gene finding. *Bioinformatics*, **24**, 637-644.

http://dx.doi.org/10.1093/bioinformatics/btn013

8. Nawrocki, E.P. and Eddy, S.R. (2013) Infernal 1.1: 100-fold faster RNA homology searches. *Bioinformatics*, **29**, 2933-2935.

http://dx.doi.org/10.1093/bioinformatics/btt509

9. Ontiveros-Palacios, N., Cooke, E., Nawrocki, Eric P., Triebel, S., Marz, M., Rivas, E., Griffiths-Jones, S., Petrov, Anton I., Bateman, A. and Sweeney, B. (2024) Rfam 15: RNA families database in 2025. *Nucleic Acids Research*, **53**, D258-D267.

http://dx.doi.org/10.1093/nar/gkae1023

10. Chan, Patricia P., Lin, Brian Y., Mak, Allysia J. and Lowe, Todd M. (2021) tRNAscan-SE 2.0: improved detection and functional classification of transfer RNA genes. *Nucleic Acids Research*, **49**, 9077-9096.

http://dx.doi.org/10.1093/nar/gkab688

11. Tremblay, B.J.-M. (2025), *yamtk: Yet Another Motif ToolKit*. 2.0.0 ed.
12. Grant, C.E., Bailey, T.L. and Noble, W.S. (2011) FIMO: scanning for occurrences of a given motif. *Bioinformatics*, **27**, 1017-1018.

http://www.ncbi.nlm.nih.gov/pubmed/21330290

http://dx.doi.org/10.1093/bioinformatics/btr064

13. Kozomara, A., Birgaoanu, M. and Griffiths-Jones, S. (2019) miRBase: from microRNA sequences to function. *Nucleic Acids Res*, **47**, D155-d162.

http://www.ncbi.nlm.nih.gov/pubmed/30423142

http://dx.doi.org/10.1093/nar/gky1141

14. Eddy, S.R. (2011) Accelerated profile HMM searches. *PLoS computational biology*, **7**, e1002195.

15. Enright, A.J., John, B., Gaul, U., Tuschl, T., Sander, C. and Marks, D.S. (2003) MicroRNA targets in Drosophila. *Genome Biology*, **5**, R1.

http://dx.doi.org/10.1186/gb-2003-5-1-r1

16. Darty, K., Denise, A. and Ponty, Y. (2009) VARNA: Interactive drawing and editing of the RNA secondary structure. *Bioinformatics*, **25**, 1974-1975.

http://www.ncbi.nlm.nih.gov/pubmed/19398448

http://dx.doi.org/10.1093/bioinformatics/btp250

17. Kerpedjiev, P., Hammer, S. and Hofacker, I.L. (2015) Forna (force-directed RNA): Simple and effective online RNA secondary structure diagrams. *Bioinformatics*, **31**, 3377-3379.

http://dx.doi.org/10.1093/bioinformatics/btv372

18. Lorenz, R., Bernhart, S.H., Höner zu Siederdissen, C., Tafer, H., Flamm, C., Stadler, P.F. and Hofacker, I.L. (2011) ViennaRNA Package 2.0. *Algorithms for Molecular Biology*, **6**, 26.

http://dx.doi.org/10.1186/1748-7188-6-26

19. Suárez-Quintana, C., Navarro-Padilla, M., Chorostecki, U. and Sanchez, M. (2025) SIREs 3.0, an improved RNA prediction tool for iron-responsive elements. *Nucleic Acids Research*, **53**, W520-W527.

http://dx.doi.org/10.1093/nar/gkaf390

20. Huang, Y., Zhang, Z., Zou, Z., Zhang, L., Chen, Y., Wan, J., Zhu, Z., Yu, S., Zuo, H., Lin, Y.-C.-D. *et al.* (2025) RegRNA 3.0: expanding regulatory RNA analysis with new features for motif, interaction, and annotation. *Nucleic Acids Research*, **53**, W485-W495.

http://dx.doi.org/10.1093/nar/gkaf405

21. Cook, K.B., Kazan, H., Zuberi, K., Morris, Q. and Hughes, T.R. (2011) RBPDB: a database of RNA-binding specificities. *Nucleic Acids Res*, **39**, D301-308.

http://www.ncbi.nlm.nih.gov/pubmed/21036867

http://dx.doi.org/10.1093/nar/gkq1069

22. Luscieti, S., Galy, B., Gutierrez, L., Reinke, M., Couso, J., Shvartsman, M., Di Pascale, A., Witke, W., Hentze, M.W., Pilo Boyl, P. and Sanchez, M. (2017) The actin-binding protein profilin 2 is a novel regulator of iron homeostasis. *Blood*, **130**, 1934-1945.

http://www.ncbi.nlm.nih.gov/pubmed/28774878

http://dx.doi.org/10.1182/blood-2016-11-754382

23. Dassanayake, R.S., Chandrasekharan, N.V. and Karunanayake, E.H. (2001) Trans-spliced leader RNA, 5S-rRNA genes and novel variant orphan spliced-leader of the lymphatic filarial nematode Wuchereria bancrofti, and a sensitive polymerase chain reaction based detection assay. *Gene*, **269**, 185-193.

http://www.ncbi.nlm.nih.gov/pubmed/11376950

http://dx.doi.org/10.1016/s0378-1119(01)00438-3
